# The first case of HIV-2 in Scotland

**DOI:** 10.1099/acmi.0.000087

**Published:** 2019-12-10

**Authors:** S. J. Shepherd, C. Sykes, C. Jackson, D. J. Bell, R. N. Gunson

**Affiliations:** ^1^​ West of Scotland Specialist Virology Centre, Level 5 New Lister Building, Glasgow Royal Infirmary, 10-16 Alexandra Parade, G31 2ER, UK; ^2^​ Infectious Diseases Unit, The Brownlee Centre, Gartnavel General Hospital, Glasgow G12 0YN, UK

**Keywords:** HIV2, antiretrovirals, diagnosis, treatment

## Abstract

HIV-1 infects an estimated 37 million people worldwide, while the rarer HIV-2 infects 1–2 million worldwide. HIV-2 is mainly restricted to West African countries. The majority of patients in Scotland are diagnosed with HIV-1, but in 2013 the West of Scotland Specialist Virology Centre (WoSSVC) diagnosed Scotland’s first HIV-2 positive case in a patient from Côte d’Ivoire. HIV-2 differs from HIV-1 in terms of structural viral proteins, viral transmissibility, prolonged period of latency, intrinsic resistance to certain antivirals and how to monitor the effectiveness of treatment. Over the course of 5 years the patient has required several changes in treatment due to both side effects and pill burden. This case highlights the complexity of HIV-2 patient management over time.

## Introduction

HIV-1 and HIV-2 were discovered in 1983 and 1986 respectively and evolved through separate zoonotic transmissions of simian immunodeficiency viruses [1]. There are eight subtypes (A to H) found in HIV-2, with subtypes A and B the most common [2]. There is also evidence that HIV-2 subtypes, like HIV-1, can exist as circulating recombinant forms [3–5]. HIV-2 is less transmissible than HIV-1 due to lower levels of viral RNA [6, 7]. While HIV-1 has spread around the world, HIV-2 has remained endemic to West Africa [8, 9]. Evidence now suggests that HIV-2 is on the decline in West Africa [10]. HIV-1 and HIV-2 dual infection can also occur [11]. Pockets of HIV-2 infection occur in other areas of the world, which shared colonial or trade routes with West Africa [12, 13]. These countries include France, Portugal, Spain and India [14–17]. There have been 183 cases of HIV-2 reported in the UK with 45 of these being dual HIV-1/HIV-2 infections [18]. This report is the first case of HIV-2 in Scotland and highlights the complexities in her management over a 5 year period.

## Case report

A 65-year-old woman, originally from Côte d’Ivoire, had been living in Scotland for 4 months, attended a GP complaining of fatigue, longstanding diarrhoea, eye pain and intermittent back pain. She had not been sexually active for several years and had no history of blood transfusion or drug misuse. It is thought that she acquired HIV through heterosexual sex.

### Diagnosis

Her routine blood work revealed a haemoglobin count of 105 g l^–1^, platelets at 134×10^9^ l^–1^, white cell count at 1.7×10^9^ l^–1^ and lymphocytes of 0.7×10^9^ l^–1^, indicating pancyotpenia and anaemia. A plasma sample was sent to the laboratory for HIV testing and the patient was found to be HIV positive on both the Abbott Architect HIV Ag/Ab combo and the bioMérieux vidas HIV Duo assays and was HIV-2 positive by the Immunocomb II HIV 1 and 2 BiSpot test. A follow-up test using the Bio-Rad Geenius HIV1/2 assay detected HIV-2 antibodies gp36 and gp140 HIV-2. A follow-up sample confirmed the initial laboratory findings. Her baseline CD4 count was 105 cells mm^−3^. She was found to be negative for HBsAg, HBV core IgG, HCV antibody, HCV antigen and syphilis. Her ophthalmic examination was normal. Plasma samples were sent to both the department of Virology at St Bartholomew’s and the London NHS Trust for HIV-2 viral-load testing and to PHE Birmingham for HIV-2 baseline resistance testing, as per the British HIV Association (BHIVA) guidelines for antiretroviral treatment of HIV-2 positive individuals (2010) [19]. Her HIV-2 viral load was detectable at 3190 copies ml^–1^ (log 3.5 copies ml^–1^) and she was HIV-2 subtype B. The patient said she was unaware of her HIV-2 status and denied taking antiretroviral therapy in the past. Her baseline resistance test (Table 1) highlighted protease inhibitor (PI) resistance mutations, with intermediate resistance to darunavir and lopinavir. Her virus was only fully susceptible to the PIs atazanavir, saquinavr and tipranavir. Her virus was susceptible to all nucleoside reverse transcriptase inhibitors (NRTI) examined and the integrase inhibitor raltegravir. The patient had a baseline drug resistance profile, which demonstrated the intrinsic nonnucleoside reverse transcriptase inhibitor (NNRTI) resistance, common to all subtypes of HIV-2 [20].

**Table 1.** HIV-2 baseline resistance report for the patient

Drug type	Probably susceptible	Possibly resistant (except NNRTI – all high level resistance)	Major resistance mutations
Protease inhibitors	Atazanavir/r Indinavir/r Saquinavir/r Tipranavir/r	Darunavir/r Lopinavir/r Nelfinavir/r	32I, 46I, 47V, 64V, 99F
Nucleoside reverse transcriptase inhibitor	Lamivudine Abacavir Zidovudine Stavudine Didanosine Emtricitabine Tenofovir		69 N, 75I
Non-nucleoside reverse transcriptase inhibitor		Delavirdine Efavirenz Etravirine Nevirapine	106I, 179T, 181V, 188L, 190A
Integrase inhibitor*	Raltegravir		T66A

*No sequencing information was supplied for integrase inhibitors Dolutegravir or Elvitegravir

### Treatment

In September 2013, she was commenced on Truvada (tenofovir/emtricitabine) and saquinavir with boosted ritonavir. Her viral load became undetectable within 1 month of starting therapy and her CD4 count rose from 105 to 133 cells mm^–3^. However, over the course of the next few years the patient required several switches in treatment due to adverse reactions (Table 2). These included prolongation of her QTc interval and palpitations thought to be due to saquinavir resulting in a switch to darunavir. She developed gastrointestinal disturbances, which she attributed to Truvada and this was changed to Combivir (lamivudine and zidovudine). The NRTI regime was switched again in June 2018 to tenofovir alafenamide (TAF) due to declining eGFR (42 ml min^−1^). To intensify her treatment and try to improve CD4 count, raltegravir was added soon after starting treatment in 2013. Raltegravir was switched to another integrase inhibitor, dolutegravir in 2018. Throughout the course of her treatment and these changes to her antivirals, the patient has continued to maintain an undetectable viral load but her CD4 count has always remained <350 cells mm^–3^. The patient was commenced on co-trimoxazole prophylaxis until her CD4 count was above 200 cells mm^–3^. The patient did not develop any opportunistic infections during this period of low CD4 count and has remained well since.

**Table 2.** Patient drug regime from September 2013 to June 2018

Regimen drug groups	Regimen	Dates	Reason for switch to next regime	CD4 count cells cmm^−1^
2xNRTI+boosted PI	Tenofovir/Emtricitabine* Saquinavir Ritonavir	Sept 2013–Nov 2013	Tenofovir/emtricitabine stopped due to patient attributed diarrhoea	105
2xNRTI+integrase inhibitor+boosted PI	Lamivudine/Zidovudine* Raltegravir Saquinavir Ritonavir	Nov 2013–Dec 2013	Raltegravir added to intensify treatment	133
2xNRTI+integrase inhibitor+boosted PI	Lamivudine/Zidovudine* Raltegravir Darunavir Ritonavir	Dec 2013–May 2017	Saquinavir stopped due to prolongation of QTc	136
2xNRTI+integrase inhibitor	Lamivudine/Zidovudine* Raltegravir	May 2017–June 2018	Darunavir and Ritonavir stopped due to high pill burden and patient stable with suppressed virus	316
2xNRTI+integrase inhibitor	Tenofovir alafenamide/Emtricitabine* Dolutegravir	June 2018	Declining eGFR. Lamivudine/Zidovudine stopped Current regimen	272

*Some of the drug regimes are available as single tablet combinations Tenofovir/emtricitabine (Truvada), Lamivudine/Zidovudine (Combivir), Tenofovir alafenamide/Emtrcitabine (Descovy).

## Discussion

This was the first HIV-2 infection diagnosed in Scotland. The patient was found to be infected with HIV-2 subtype B, which is the most prevalent HIV-2 subtype in Côte d’Ivoire where the patient originated from [1].

Our patient was initially screened for HIV1/2 using the Abbott Architect and bioMérieux Vidas assays, both commonly used fourth-generation HIV assays. Fourth-generation assays have helped to reduce the window period of detection for HIV-1 due to the addition of monoclonal antibody to detect HIV-1 p24 antigen but as this is not present in HIV-2, the window period for HIV-2 remains the same as for third-generation antibody only assays. Therefore, unlike HIV-1, HIV-2 early infections, where only viral antigen is present, will not be detected until the patient has developed HIV-2 antibodies. None of the available fourth-generation assays can distinguish between HIV-1 and HIV-2. Therefore, a confirmation antibody assay is routinely required to differentiate between HIV-1 and 2. The patient in this study was confirmed as being HIV-2 positive using the ImmunoComb II HIV 1 and 2 BiSpot. However, because there is a 50–60 % amino acid sequence similarity between HIV-1 and HIV-2 cross-reactivity can occur with HIV 1/2 confirmation assays. This cross-reactivity has been seen in all HIV-1/2 typing assays, including HIV-1 Western blot assays. Such cross-reactivity can result in a patient being wrongly identified as an early/indeterminate HIV-1 positive or as a HIV-1/HIV-2 dual infection [21–24].

There is a strong correlation between HIV-2 plasma viral load and mortality rate [25]. HIV-2 tends to run a less aggressive course than HIV-1, with a more prolonged latent period characterized by a lower or undetectable viral load [26, 27]. The mortality rate is three to sixfold lower in HIV-2 compared to HIV-1. However, in patients where the HIV-2 viral load is detected at ≥10 000 copies ml^−1^ the mortality rate is similar to that seen in HIV-1 infections [25, 28]. There are key amino acids changes in the capsid protein of HIV-2, which may lead to higher viral load in some patients [29]. Unlike HIV-1, there are no commercial HIV-2 viral-load assays and variation exists between in-house assays [21, 30]. The measurement of HIV-2 viral load is only available in a few centres in the UK. At the time of diagnosis the patient had a viral load of 3190 copies ml^−1^, confirming her HIV-2 status. The HIV-2 viral load became undetectable once treatment commenced. Plasma viral load and CD4 count are independent but significant predictors of mortality in HIV-2 infection [28]. In keeping with longstanding infection the patient had a low-level CD4 count (105 cells mm^–3^) and with treatment the CD4 count peaked at 323 cells mm^–3^. This low baseline CD4 count suggests she had been infected for a long time period prior to diagnosis and therefore immune reconstitution is poorer in these patients once treatment has started [31, 32].

Due to its worldwide prevalence, there is an in-depth knowledge of HIV-1 resistance [33]. However, this is not the case for HIV-2. Polymorphic variation exists within both HIV-1 and HIV-2 and can result in resistance to antivirals. However, the effect the polymorphism has on antiviral treatment will vary depending on whether it is HIV-1 or HIV-2 [34, 35]. For example, due to natural polymorphisms at amino acid residues 181 and 188 in the reverse transcriptase gene, HIV-2 is resistant to all NNRTIs. Resistance testing is important because it can not only detect these natural polymorphisms but can also detect mutations occurring through viral replication when on treatment. HIV-2 is said to have a low barrier of resistance to NRTIs [36]. A West African collaboration (IeDEA-WA-HIV-2 cohort) has helped to implement an HIV-2 and HIV-1/HIV-2 drug-resistance database [37]. At the time of diagnosis in 2013, there were around 180 known cases of HIV-2 infection in the UK, although this was the first in Scotland. Her treatment was informed by reference to the British HIV Association 2010 Guidelines on the management of HIV-2 and after helpful discussion with an expert at St Bartholomew’s and the London NHS Trust who runs a clinical service for HIV-2 infected patients [18, 19].

The patient was found to be susceptible to all NRTI drugs and was started on two NRTIs and boosted PI regimen. A study of HIV-2 patients found superior viral-load suppression and CD4 cell recovery, as well as improved clinical outcomes, if a PI containing regime was used in combination with NRTIs [38]. *In vitro* evidence suggests that PI drugs lopinavir, darunavir and saquinavir, all boosted with ritonavir, have an inhibitory effect on HIV-2 comparable to that in HIV-1. The patient was initially started on ritonavir boosted saquinavir, to which she was fully susceptible. Unfortunately, the patient developed a prolonged QTc interval on her ECG on saquinavir, increasing from 436 to 474 ms. This is a well described side effect of saquinavir and one reason why it no longer a first line PI in HIV-1 treatment [39]. The decision was taken to switch her PI from saquinavir to darunavir. Her baseline profile indicated probable resistance to darunavir, but given the complexity of HIV-2 resistance interpretation and limited PI options, the change in PI was made.

The patient was started on the NRTIs tenofovir disoproxil fumarate and emtricitabine (Truvada). This was changed to lamivudine and zidovudine (Combivir) when the patient attributed gastrointestinal disturbances to the Truvada. The NRTI regime was again switched in June 2018 to tenofovir alafenamide and emtricitabine (Descovy) due to declining renal eGRF [40]. Initially the patient was commenced on raltegravir and then switched to dolutegravir in 2018. Integrase inhibitors appear to be effective in HIV-2. However, natural polymorphisms conferring resistance to raltegravir have been found in integrase treatment naïve patients [41, 42]. Dolutegravir is a second-generation integrase inhibitor, which in HIV-1 may be effective even in the presence of some raltegravir resistance mutations.

### Conclusion

This is the first case of HIV-2 in Scotland. This patient receives regular clinical checks as per all HIV-positive patients in Scotland. Most patients living with HIV in the UK are now on anti-retroviral therapy in order to prevent disease progression. Since starting on treatment, her HIV-2 viral load has remained undetectable and her CD4 count has risen from an initial 105 cells mm^–3^ to a peak of 323 cells mm^–3^ . This modest rise in CD4 count since starting on treatment reflects her low baseline CD4 count. Since this case, a further HIV-2 positive patient was diagnosed by WoSSVC in 2017. This patient was also originally from West Africa. More patients with HIV-2 infection are likely to present in the future as people move to Scotland from elsewhere in the UK or abroad. For this reason, laboratories and clinicians need to be aware of the limitations of current laboratory assays and the specialist testing and interpretation required for resistance profiling of HIV-2. This case should serve as a timely reminder of the complexity of both diagnosis and patient management.
